# In vitro differentiation of W8B2^+^ human cardiac stem cells: gene expression of ionic channels and spontaneous calcium activity

**DOI:** 10.1186/s11658-020-00242-9

**Published:** 2020-11-05

**Authors:** Oualid Ayad, Zeina R. Al Sayed, Stéphane Sebille, Christophe Magaud, Charles-Albert Chapotte-Baldacci, Christophe Jayle, Jean-François Faivre, Nathalie Gaborit, Aurélien Chatelier, Patrick Bois

**Affiliations:** 1 University of Poitiers Signalisation et Transports Ioniques Membranaires, EA7349, Poitiers Cedex 09, France; 2 CHU of Poitiers chirurgie cardiaque et thoracique, , Poitiers Cedex 09, France; 3 CNRS, INSERM, l’institut du thorax, Université de Nantes, 44000 Nantes, France

**Keywords:** W8B2^+^ human cardiac stem cell, Cardiac differentiation, Calcium activity, Oscillations, Ion channels, GCaMP

## Abstract

**Background:**

Human cardiac stem cells expressing the W8B2 marker (W8B2^+^ CSCs) were recently identified and proposed as a new model of multipotent CSCs capable of differentiating into smooth muscle cells, endothelial cells and immature myocytes. Nevertheless, no characterization of ion channel or calcium activity during the differentiation of these stem cells has been reported.

**Methods:**

The objectives of this study were thus to analyze (using the TaqMan Low-Density Array technique) the gene profile of W8B2^+^ CSCs pertaining to the regulation of ion channels, transporters and other players involved in the calcium homeostasis of these cells. We also analyzed spontaneous calcium activity (via the GCaMP calcium probe) during the in vitro differentiation of W8B2^+^ CSCs into cardiac myocytes.

**Results:**

Our results show an entirely different electrophysiological genomic profile between W8B2^+^ CSCs before and after differentiation. Some specific nodal genes, such as Tbx3, HCN, ICaT, L, KV, and NCX, are overexpressed after this differentiation. In addition, we reveal spontaneous calcium activity or a calcium clock whose kinetics change during the differentiation process. A pharmacological study carried out on differentiated W8B2^+^ CSCs showed that the NCX exchanger and IP3 stores play a fundamental role in the generation of these calcium oscillations.

**Conclusions:**

Taken together, the present results provide important information on ion channel expression and intrinsic calcium dynamics during the differentiation process of stem cells expressing the W8B2 marker.

## Background

The existence in healthy and pathological human cardiac tissue of rare populations of multipotent cardiac stem cells (CSCs) that are capable of differentiating into myocytes, smooth muscle cells and endothelial cells has been demonstrated by several groups [1–4]. Several types of CSCs have been isolated and characterized based on the expression of specific markers, including c-kit^+^ CSCs, Isl-1^+^ CSCs, SP (Side Population) CSCs and Sca-1^+^ CSCs [5]. CSCs can be used in autologous cell transplantation for cardiomyocyte replacement in patients with cardiac disorders such as myocardial infarction [6–8].

A new population of CSCs expressing the W8B2 marker (W8B2^+^ CSCs) has been characterized, where W8B2 or MSCA-1 (mesenchymal stem cell antigen-1) is a tissue non-specific alkaline phosphatase (TNAP) [9]. Aguiar et al. [10] first demonstrated that these cells were of mesenchymal origin, although genomic analysis later showed that these cells differ in their gene expression from bone marrow-derived mesenchymal stem cells and from c-kit^+^ CSCs [11, 12]. In addition, W8B2^+^ CSCs express cardiac-specific transcription factors such as GATA4 and can be differentiated into endothelial cells, smooth muscle cells and immature myocytes lacking mature contractile structures [12]. To date, myocytes derived from cardiac stem cells after in vitro differentiation have exhibited immature functional and structural characteristics compared to native adult cardiomyocytes. A future challenge will thus be to develop strategies to improve myocyte maturation.

W8B2^+^ CSCs possess significant cardiac repair capacities when injected into myocardial infarction mouse models [11, 12]. This cardiac repair capacity is related to the large secretome (composed of cytokines and growth factors) of W8B2^+^ CSCs, which is implicated in angiogenesis, cell survival, chemotaxis, immune response, inflammation and extracellular remodeling [12, 13]. It was recently shown that W8B2^+^ CSCs also secrete exosomes containing cargo proteins, mRNAs and pre-microRNAs capable of modulating several cellular pathways involved in protein metabolism and cell growth as well as cellular responses to stress and organization of the extracellular matrix [14]. Despite what is currently known, no functional characterization of W8B2^+^ CSCs at the cellular level (electrophysiology, calcium signaling) during cardiac differentiation has been reported. Indeed, very little information on the expression of ion channels and calcium activity during cardiac differentiation in human CSCs is available.

We and others have shown that ion channels (including potassium channels) regulate the proliferation of both human W8B2^+^ CSCs [15] and human bone marrow-derived MSCs [16]. Interestingly, inhibition of BKCa or hEag1 channels reduced the adipogenic and osteogenic differentiation of these cells [16]. In addition, inhibition of L-type Ca^2+^ channels reduced the differentiation of neural progenitor cells derived from the mouse cerebral cortex [17]. Another study showed that human c-kit^+^ CSCs exhibit spontaneous calcium oscillatory activity, which plays a key role in the regulation of cell proliferation [18]. These data suggest that ion channels may play a crucial role in the cardiac differentiation process. We also consider that calcium oscillations might also be involved in cardiac differentiation, making it important therefore to study their existence, fate, evolution and involvement in this process.

Through our targeted transcriptomics against the genes encoding ion channels and the calcium functional study, we hope to identify key players whose expression, or lack thereof, might be responsible for this immature differentiation. In addition, monitoring calcium activity, the parameters of which seem to be modified during differentiation, could be a spatiotemporal tool for following the fate of MSCs.

The objective of this study was therefore to characterize the gene profile of W8B2^+^ CSCs (using the TaqMan Low-Density ArrayTLDA technique) pertaining to ion channels and calcium homeostasis after the induction of cardiac differentiation and to monitor calcium changes during differentiation with the aid of the GCaMP calcium probe, a genetically encoded calcium indicator.

## Materials and methods

### Isolation of W8B2^+^ CSCs

Human W8B2^+^ CSCs were isolated as previously described (Ayad et al. 2017). Human right atrial tissue specimens were obtained from 7 adult patients (mean age 77 ± 5.7 years, 6 males and 1 female). These tissue samples are surgical waste resulting from the implementation of extracorporeal cardiac surgery, such as coronary artery bypass surgery, and were obtained in cooperation with the University Hospital of Poitiers. All procedures were carried out in accordance with the Declaration of Helsinki. Briefly, freshly harvested specimens were manually minced into 1–2 mm^3^ fragments and subjected to enzymatic digestion with collagenase A (1 mg/mL, Sigma-Aldrich) for 20 min at 37 °C. The tissue fragments were plated on fibronectin-coated dishes (10 μg/mL, Roche Diagnostics) and cultured in medium explant containing IMDM medium (Lonza) supplemented with 20% fetal bovine serum (Biowest), 0.1 mM β-mercaptoethanol (Sigma-Aldrich), 2 mM l-glutamine, 100 μg/mL streptomycin (Sigma-Aldrich), 100 μg/mL penicillin (Sigma-Aldrich) and 0.25 μg/mL amphotericin B (Sigma-Aldrich) at 37 °C and 5% CO_2_. After 2–3 weeks, monolayers of adherent cells were partially enriched for the W8B2 marker using a magnetic cell sorting system (Miltenyi Biotec, Bergisch Gladbach, Germany) to identify W8B2 antibody-tagged cells. The positive fraction (W8B2^+^ cells) was seeded in growth medium containing 25% EGM-2 (Lonza) and 75% M199 (Lonza) supplemented with 10% fetal bovine serum (Biowest), 100 μg/mL streptomycin (Sigma-Aldrich) and 0.25 μg/mL amphotericin B (Sigma-Aldrich), 0.1 mM nonessential amino acids, and 100 U/mL penicillin. Cells were cultured at 37 °C and 5% CO_2_. After magnetic cell sorting, confluent cells were labeled with W8B2 PE-conjugated antibody and released using a FACS Aria Flow cytometer and sorter using MSCA-1 (W8B2) antibody coupled to phycoerythrin (1:11, Miltenyi Biotec, Bergisch Gladbach, Germany). After cell sorting, the collected W8B2^+^ cells were seeded into flasks containing growth medium.

### Immunostaining

W8B2^+^ CSCs were seeded on gelatin-fibronectin-coated glass slides. They were then fixed, permeabilized and incubated overnight with primary antibodies directed against GATA4, Nkx2.5, MEF2C, TNNT2, a-actinin and b-MHC. Cells were washed three times with phosphate-buffered saline and incubated for 2 h with the secondary antibody. TOPRO (1:1000, Invitrogen, USA) was used for labelling of nuclei. Finally, the slides were mounted in Mowiol (Sigma-Aldrich) and cells visualized using confocal microscopy.

### In vitro differentiation

To induce in vitro cardiac differentiation, W8B2^+^ CSCs were seeded at a density of 2 × 10^4^ cells/cm^2^ on plates coated with 10 μg/ml fibronectin (Sigma-Aldrich) and 0.1% gelatin (Sigma-Aldrich) in growth medium. After overnight incubation, cells were treated with 5-azacytidine (10 μM; Sigma-Aldrich) in the growth medium for 24 h and then cultured in differentiation medium (containing IMDM medium (Lonza) supplemented with 10 nM dexamethasone, 5 μM phenylbutyrate and 5 μM retinoic acid). The differentiation medium was renewed every 2 days.

### RNA preparation

Extraction of total RNA from W8B2^+^ CSCs and differentiated W8B2^+^ CSCs (specimens obtained from 4 adult patients, mean age 75.2 ± 9.1 years, 3 males and 1 female) was performed with the RNA Extraction Kit (NucleoSpin Macherey–Nagel). Cells were collected and lysed with RA1 buffer and β-mercaptoethanol. The lysate was then filtered with (NucleoSpin Filter) columns and centrifuged (1 min at 11,000*g*). After filtration, 70% ethanol was added to the lysate and the mix was deposited on columns (NucleoSpin RNA Column). The lysate was then centrifuged (30 s at 11,000*g*). After adding Membrane Desalting Buffer (Sigma-Aldrich) the lysate was centrifuged (1 min at 11,000*g*) to remove salts. The DNA was then digested with DNase reaction mixture for 15 min at room temperature and RNA rinsed with RAW2 and RA3 buffers. Finally, the RNA was eluted in 60 μL of water (RNase-free water) and centrifuged (1 min at 11,000*g*). The RNA concentration in each sample was measured using the NanoDrop Spectrophotometer ND-1000 (NanoDrop Technologies Inc, USA).

### TaqMan real-time reverse transcriptase-polymerase chain reaction

The TaqMan low-density array (TLDA, Applied Biosystems) technology was used in a two-step reverse transcription polymerase chain reaction (RT-PCR) process. First-strand cDNA was synthesized from total RNA using a reverse transcription kit (Super Script IV VILO, Invitrogen). For this purpose, 16 μL of total RNA (containing 1 μg of total RNA) were added to 4 μL of superscript IV VILO Master mix. After rapid centrifugation, the mixture was incubated for 10 min at 25 °C, followed by 20 min at 50 °C and finally 5 min at 85 °C. PCR reactions were then performed on a TLDA fitted with the ABI Prism 7900HT Sequence Detection System (Applied Biosystems). TaqMan master mix was added to the 384 wells of the TaqMan Array Micro Fluidic Card, AB preloaded with TaqMan fluorescent hydrolysis probes and primers. The amplification was carried out over forty cycles (each cycle included a denaturation step for 30 s at 97 °C and a hybridization and elongation step for one minute at 59.7 °C). Data were collected using Applied Biosystems SDS 2.1 software and analyzed with the threshold cycle (Ct) relative-quantification method (Livak and Schmittgen 2001). The 96 human genes selected for gene expression encode potassium, calcium, sodium and HCN channels, proteins involved in signaling and calcium homeostasis, connexins, transcription factors, cardiac structural proteins, natriuretic peptide (NP) and four reference genes for normalization. We selected the Ribosomal Protein L13a (RPL13A) gene for data normalization as the most uniformly distributed gene. The relative expression of each gene was calculated for each sample (ΔCt (delta between Ct) indicates standardized data). The expression variation of each gene in differentiated W8B2^+^ CSCs versus undifferentiated W8B2^+^ CSCs is represented by the −ΔΔCt (double delta Ct) formula.

### Calcium imaging

W8B2^+^ CSCs (specimens obtained from 6 adult patients (mean age 75.8 ± 6.2 years, all male) were seeded at a density of 2 × 10^4^ cells/cm^2^ in 35 mm glass bottom dishes coated with fibronectin (10 μg/mL) and gelatin (0.1%) in the growth medium. The next day, cells were infected with 4 × 10^9^ viral particles/ml of AAV1.CAG.GCaMP6s calcium probe for 12 h in growth medium and then differentiated as described above. pAAV.CAG.GCaMP6s.WPRE.SV40 was a gift from Douglas Kim & GENIE Project (UPen, Baohan et al. 2013) and AAV2/1 were produced at the Canadian Neurophotonics Platform during differentiation (from day 2 to day 28). Spontaneous calcium activity (corresponding to fluorescence emitted by GCaMP excited at 488 nm) was recorded using a confocal microscopy rotating disc (Olympus IX81-ZDC, Andor) equipped with an incubation system (at 37 °C and 95% air-5% CO_2_). Calcium recordings were performed at 40× magnification for 10 min at one image/second. The pharmacological study was performed using an infusion device (made in house) allowing the administration of pharmacological agents during the recording. Raw calcium records were analyzed using a "macro" applied using Image J software that quantified overall fluorescence changes in each cell studied.

For graphs representing calcium activity (in the form of oscillations), fluorescence variations were normalized by applying the formula ΔF/F0 (where ΔF represents the variation of fluorescence intensity (FI) at time *t* relative to a basal fluorescence value (F0) obtained by averaging the lowest fluorescence values). The analysis of calcium oscillation parameters [frequency, peak amplitude, duration and time to peak (TTP)], was performed using a program developed in our laboratory from IDL software. The peak amplitude [expressed in arbitrary units (AU)] corresponds to the maximum ratio ΔF/F0, the frequency (expressed in peaks/min) was estimated over 10 min, the duration (expressed in seconds) was measured at the mid-height of the curve, and TTP (expressed in seconds) corresponds to the time required to pass from the basal fluorescence F0 to the maximum fluorescence F. The evolution of these parameters is represented according to week for the four weeks of differentiation: the first week taking into account all calcium oscillations obtained between the 2nd and 7th days after differentiation, the second week taking into account all calcium oscillations obtained between the 8th and 14th days after differentiation, the third week taking into account all calcium oscillations obtained between the 15th and 21st days after differentiation, and finally the fourth week taking into account all calcium oscillations obtained between the 22nd and 28th days after differentiation.

### Statistical analysis

Results were expressed as mean ± SEM. All statistical analyses were performed using GraphPad Prism (La Jolla, CA, USA). Statistical tests used for the different assays are provided in the figure legends.

## Results

### Transcriptomic profile of cardiac markers after in vitro differentiation of W8B2^+^ CSCs

**Fig. 1:**
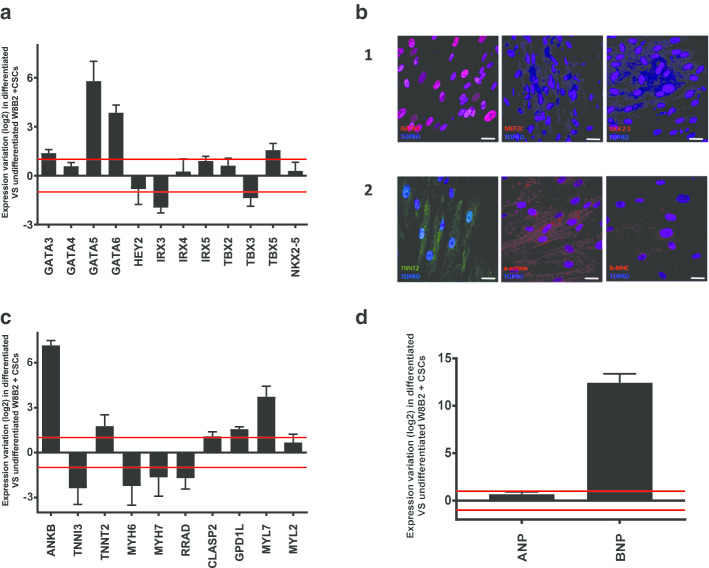
Effect of W8B2^+^ CSCs in vitro differentiation on cardiac markers. **a** Transcript coding for cardiac transcription factors analyzed by TLDA in differentiated vs undifferentiated W8B2^+^ CSCs. **b** Determination by immunostaining of protein expression profile of GATA4, MEF2C and NKX 2.5 (**b1**) and troponin T, a-actinin and b-MHC (b2) after in vitro differentiation of W8B2^+^ CSCs. Nuclei are represented in blue with TOPRO staining. Scale bar 100 μm. **c** Transcript coding for cardiac contractile structures analyzed by TLDA in differentiated vs undifferentiated W8B2^+^ CSCs. **d** Transcript coding for natriuretic peptides [atrial (ANP) and brain (BNP)] analyzed by TLDA in differentiated vs undifferentiated W8B2^+^ CSCs. The graphs illustrate the gene expression variations (expressed in log 2) of the differentiated cells versus the undifferentiated cells. Values between 1 and − 1 (horizontal red lines) indicate that gene expression does not vary (this corresponds to variations of + 2 times and − 2 times compared to undifferentiated cells). Each bar represents the mean value ± S.E.M. (standard error of the mean). (N = 4 patients)

Cardiac transcription factors, which are essential for cardiac development and morphogenesis studies, were selected to characterize W8B2^+^ CSCs prior to differentiation and then for the evolution of these factors after 28 days of in vitro differentiation. Results illustrated in Fig. 1a show that the transcripts coding for GATA5 and GATA6 were strongly increased (55-fold increase for GATA5 and 15-fold increase for GATA6) in differentiated versus undifferentiated CSCs. The expression of GATA3 and TBX5 was slightly increased (2.5-fold for GATA3 and threefold for TBX5), while no variation of gene expression for the transcription factors GATA4, HEY2, IRX4, IRX5, TBX2 and NKX2-5 was observed. Regarding the transcription factors IRX3 and TBX3, a slight reduction of their transcripts was detected (fourfold decrease for IRX3 and 2.5-fold decrease for TBX3). At the protein level, immunostaining results indicated that differentiated W8B2^+^ CSCs expressed the GATA4 transcription factor, but not MEF2C and NKX2.5 (Fig. 1b1).

To investigate cardiac differentiation, several specific markers were analyzed after employing an in vitro cardiac differentiation protocol. The most-used markers for this purpose are cardiac transcription factors, contractile structures and secreted factors. We examined gene expression levels of markers such as troponin, myosin, ankyrin and natriuretic peptides. The results obtained by TLDA (Fig. 1c) display strong induction of the expression of the transcripts encoding ANKB and MYL7 during cardiac differentiation (137-fold increase for ANKB and 13-fold increase for MYL7). Slight induction was observed for TNNT2 and GPD1L (threefold increase for TNNT2 and threefold increase for GPD1L). No changes in expression were seen for CLASP2 and MYL2. On the other hand, the expression of transcripts coding for TNNI3, MYH6, MYH7 and RRAD was slightly repressed during the differentiation process (5 -fold decrease for TNNI3, fourfold decrease for MYH6, threefold decrease for MYH7 and threefold decrease for RRAD). Immunostaining also indicated that there were no mature contractile structures (absence of typical cardiomyocyte striations) as evidenced by the labeling of TNNT2, α-actinin and b-MHC (Fig. 1b2). Regarding the expression of natriuretic peptides, the results obtained showed very strong induction (5500-fold increase) of BNP whereas the expression of ANP remained unchanged (Fig. 1d).

### Spontaneous calcium activity changes during in vitro differentiation of W8B2^+^ CSCs

**Fig. 2:**
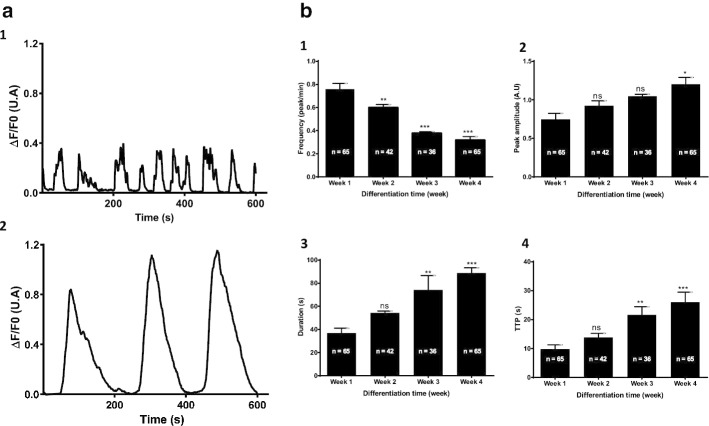
Evolution of spontaneous calcium activity parameters during W8B2^+^ CSC differentiation. **a** Types of spontaneous calcium activity recorded at the beginning of the differentiation process [day 2 after differentiation (1)] and at the end of the differentiation process [day 28 after differentiation (2)]. The graphs illustrate variations in [Ca^2+^]_i_ represented by ΔF/F0 ratio, where F0 represents the basal fluorescence emitted by the GCaMP, and ΔF the difference between basal and actual fluorescence (F) at a given time point. **b** Bar graphs (1), (2), (3), (4) represent the evolution of the frequency, the peak amplitude, the duration and TTP (time to peak) of calcium oscillations, respectively, recorded during the 4 weeks of W8B2^+^ CSC differentiation. (N = 6 patients, n: represents the number of cells analyzed, AU: arbitrary unit, ns: not significant). *p < 0.05, **p < 0.01, ***p < 0.001 vs. week 1; one-way ANOVA followed by a Newman-Keuls test

To our knowledge, initial calcium activity and temporal changes during W8B2^+^ CSC differentiation have not been reported to date. To address this knowledge gap, calcium activity during the in vitro differentiation of W8B2^+^ CSCs was assessed using the GCaMP6s calcium probe as a dynamic sensor of intracellular calcium concentration changes. Calcium activity was recorded by confocal microscopy at 37 °C and 95% air-5% CO_2_ for four weeks (corresponding to the time of the differentiation protocol), with fluorescence variations presented as time-dependent curves. The results in Fig. 2 show for the first time the presence of spontaneous calcium activity in W8B2^+^ CSCs. At the beginning of differentiation, calcium activity appears as irregular oscillations (Fig. 2a1), with a higher frequency and lower peak amplitude than those observed at the end of the differentiation process (Fig. 2a2). Note that during the latter stage of differentiation, the calcium oscillations become much slower and more regular. At this stage, no spontaneous contraction was observed. Different kinetic parameters of the calcium oscillations were obtained, such as the peak amplitude, frequency, duration and TTP (Fig. 2b). The oscillation frequency decreased significantly (by ~ 60%) over the 4-week differentiation period (from 0.75 peak/min in week 1 to 0.31 peak/min in week 4 of differentiation) (Fig. 2b1). This decreasing frequency was also significant when comparing week 1 to week 2 (a 20% decrease) or week 1 to week 3 (a ~ 51% decrease).

The peak amplitude of calcium oscillations, which provides information concerning the level of intracellular calcium release and/or activity, increased progressively, with the difference at week 4 with respect to week 1 reaching statistical significance (Fig. 2b2). In this way, the peak amplitude increased by ~ 72%, from 0.7 AU in week 1 to 1.2 AU at week 4.

Analysis of the duration of calcium oscillations (Fig. 2b3) indicated a gradual increase in duration over the differentiation period. The average duration was ~ 40 s in week 1 compared to ~ 90 s in week 4 (141% increase). Differences were also significant between week 1 (~ 37 s) and week 3 (~ 75 s) (~ 103% increase).

TTP corresponds to the time between the onset of increased intracellular calcium release and the time for peak concentration to be reached within a single oscillation. Figure 2b4 shows that TTP increased gradually, from ~ 10 s in week 1 to 26 s in week 4 (~ 160% increase). The increase from post-differentiation week 1 to week 3 was also significant (~ 120% increase).

### Transcriptomic profile of excitation–contraction coupling markers after in vitro differentiation of W8B2^+^ CSCs

**Fig. 3:**
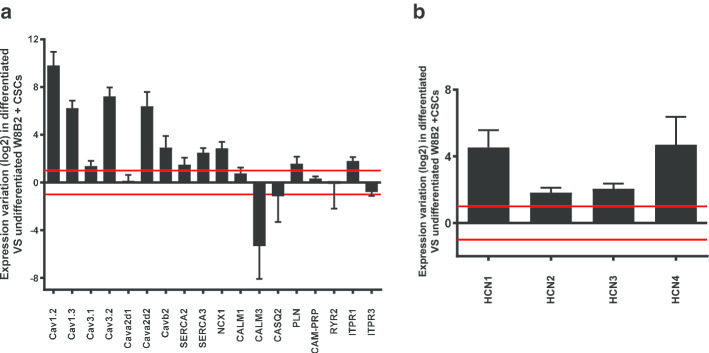
Effect of W8B2^+^ CSC in vitro differentiation on calcium channels, ion exchangers and contributors to calcium homeostasis. Transcript coding for players in the process of calcium homeostasis analyzed by TLDA in differentiated vs undifferentiated W8B2^+^ CSCs. Graphs illustrate gene expression variations (in log 2) of differentiated cells versus undifferentiated cells from N = 4 patients. Horizontal red lines correspond to 1 and − 1 values

To identify the molecular mechanisms involved during W8B2^+^ CSC differentiation, a study of the transcriptomic profile of excitation–contraction coupling markers was carried out. In the heart, L-type voltage-gated calcium channels (Cav) are among the major players responsible for the cardiac action potential as well as in the calcium-induced calcium release process, which facilitates cardiac muscle contraction. On the other hand, the T-type calcium channel is important in depolarization events and participates in the rhythmic activity of the sinoatrial node. The transcript expression analysis after W8B2^+^ CSC differentiation showed strong induction of transcripts coding for L-type Cav channels: Cav1.2 (900-fold increase), Cav1.3 (75-fold increase) (Fig. 3a). The transcripts coding for T-type Cav are differentially expressed: Cav3.2 (150-fold increase) and slight induction of Cav3.1 (2.5-fold increase). Concerning channel regulatory subunits, we observed augmented expression of Cava2d2 (85-fold increase), Cavb2 (7.5-fold increase), and no change in Cava2d1 expression.

With respect to ion exchangers and pumps, our results showed a significant increase in the expression of transcripts coding for the sarco-endoplasmic reticulum calcium pumps (Ca^2+^-ATPase) SERCA3 (sixfold increase) and the NCX1 exchanger (sevenfold increase). Note the non-significant induction of SERCA2. In addition, the transcript coding for calmodulin 3 (CALM3) was highly repressed (40-fold decrease) while that of calmodulin 1 (CALM1) was not changed during the differentiation process. We also observed slight induction of IP3 receptors (ITPR1; 3.5-fold increase), while non-significant variation was detected in the expression of transcripts coding for phospholamban (PLN), CALM1, calsequestrin (CASQ2), calmodulin-dependent protein phosphatase (CAM-PRP), ryanodine receptor (RYR2) and ITPR3.

HCN channels in nodal cells are responsible for the pacemaker current underlying the genesis of cardiac rhythm. To verify whether the differentiation process could trigger a nodal cell phenotype, expression levels of HCN channels were assessed. The results illustrated in Fig. 3b show strong induction of HCN1 (23-fold increase) and HCN4 (25-fold increase) channel transcripts and weaker induction of HCN2 (3.5-fold increase) and HCN3 (fourfold increase) transcripts.

### Identification of players involved in the generation of calcium oscillations in differentiated W8B2^+^ CSCs

**Fig. 4:**
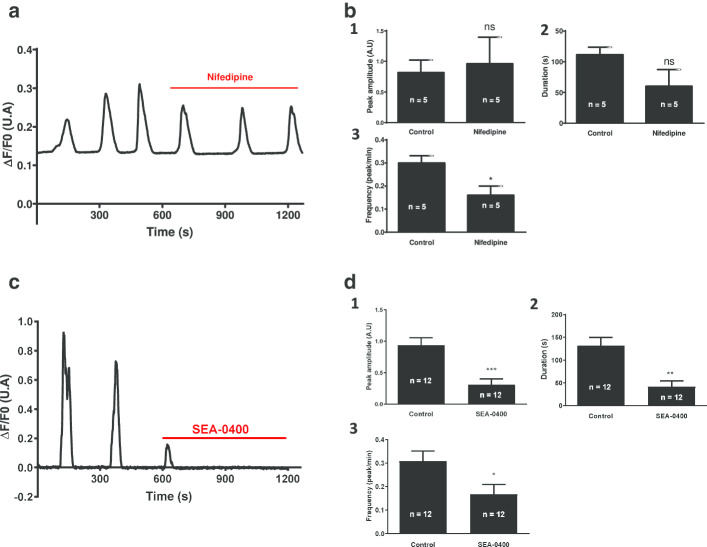
Role of L-type calcium channel and NCX exchanger activity in W8B2^+^ CSCs after 28 days of in vitro differentiation. **a** Effect of 5 µM nifedipine (L-type CaV inhibitor) and **c** 3 µM SEA-0400 (NCX exchanger inhibitor) on spontaneous calcium activity recorded in differentiated CSCs. Graphs illustrate variations in [Ca^2+^]_i_ as represented by changes in the ΔF/F0 ratio. ΔF = F − F0 (F: fluorescence emitted by CaMP at a given time; F0: basal fluorescence emitted by GCaMP). Bar graphs (1), (2), (3) represent the evolution of the peak amplitude, the duration and frequency of calcium oscillations, respectively, recorded from differentiated W8B2^+^ CSCs after treatment with nifedipine (5 µM) (**b**) and SEA (3 µM) (**d**). (N = 3 patients, n: represents the number of events (oscillations) recorded, AU: arbitrary unit, ns: not significant). *p < 0.05, **p < 0.01, ***p < 0.001 vs. control; t-test paired followed by a Wilcoxon test

**Fig. 5:**
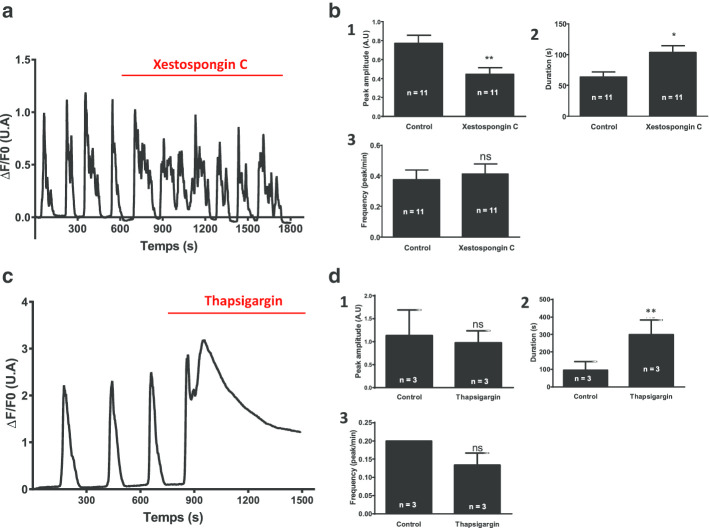
Role of SERCA and IP3 receptors on spontaneous calcium activity recorded in W8B2^+^ CSCs after 28 days of in vitro differentiation. **a** Effect of 5 µM xestospongin C (IP3 receptor inhibitor) and **c** 1 µM thapsigargin (SERCA inhibitor) on spontaneous calcium activity recorded in differentiated CSCs. Graphs illustrate variations in [Ca^2+^]_i_ represented by changes in the ΔF/F0 ratio. ΔF = F − F0 (F: fluorescence emitted by GCaMP at a given time; F0: basal fluorescence emitted by GCaMP). Bar graphs (1), (2), (3) represent the evolution of the peak amplitude, the duration and the frequency of calcium oscillations, respectively, recorded from differentiated W8B2^+^ CSCs after treatment with xestospongin C (5 µM) (**b**) and thapsigargin (1 µM) (**d**). (N = 3 patients, n: represents the number of events (oscillations) recorded, AU: arbitrary unit, ns: not significant). *p < 0.05, **p < 0.01 vs. control; t-test paired followed by Wilcoxon test

To evaluate the major players involved in calcium homeostasis and in the generation of calcium oscillations observed in differentiated W8B2^+^ CSCs (after 28 days of in vitro differentiation), we used pharmacological agents to inhibit activities of the NCX exchanger (SEA0400), SERCA (thapsigargin), IP3 receptors (xestospongin C), L-type calcium channels (nifedipine) and ryanodine receptors (high-concentrations of ryanodine). The effects of these inhibitors on calcium oscillations were analyzed and the results are shown in Figs. 4 and 5.

The addition of 5 μM of nifedipine (L-type calcium channel blocker) changed the cytoplasmic calcium concentration, inducing smaller, sustained fluctuations (Fig. 4a). Note that only the oscillatory frequency was significantly reduced (Fig. 4b). The perfusion of SEA0400 (3 μM), a selective inhibitor of the NCX1 exchanger, substantially reduced the calcium oscillations observed in the differentiated W8B2^+^ CSCs (Fig. 4c). All measured parameters (peak amplitude, duration, frequency) were decreased (Fig. 4d).

The addition of xestospongin C (5 μM; a specific inhibitor of IP3 receptors) (Fig. 5a) did not impact on the occurrence of the calcium oscillations but induced a significant decrease in their peak amplitude (by ~ 43%) (Fig. 5b1), and a significant increase (by ~ 64%) in the duration of slow oscillations (Fig. 5b2) without modifying their frequency (Fig. 5b3).

The application of 1 μM of thapsigargin was followed by the appearance (in this example) of calcium oscillation interrupted by a new calcium elevation that gradually decreased (Fig. 5c). Only the duration was significantly increased (Fig. 5d2).

The addition of ryanodine 50 μM (a concentration that inhibits the RyR) did not induce any change in the peak amplitude, frequency or duration of the calcium oscillations. As expected, this result corroborates the lack of expression of the ryanodine receptor at the transcriptomic level on W8B2^+^ CSCs prior to differentiation (data not shown).

## Discussion

### Transcriptomic profile of cardiac markers after W8B2^+^ CSC differentiation

Adult stem cells are able to differentiate in vivo and generate new, specialized cells of the tissue in which they are found [19, 20]. In the present study, we focused on the cardiac differentiation capacity of W8B2^+^ CSCs, the results of which could prove important, especially for regenerative medicine employing the autologous injection of cardiac stem cells to replace necrotic tissue in patients with cardiac disorders.

To induce cardiac differentiation, we used chemical inducers based on the work of Zhang and colleagues [12]. Our results showed that after 28 days of in vitro differentiation, the W8B2^+^ CSCs presented a very different transcriptomic profile from undifferentiated cells. Nevertheless, the differentiated cells did not exhibit the characteristic phenotype of mature cardiomyocytes at the protein level (typical striations characteristic of contractile structures were absent) or transcriptomic level (no transcriptomic induction, in particular for cardiac myosin heavy chains). In contrast, some transcripts encoding for cardiac differentiation markers such as ankyrin B (ANKB), myosin light chain and brain natriuretic peptide (BNP) were overexpressed. ANKB was shown to be a regulatory protein for human sinoatrial node automatism [21, 22], probably via Cav1.3 calcium channel activity [23]. Among the myosin light chains, the MYL7 atrial isoform is overexpressed during cardiac differentiation, as shown in a previous study [12]. These results are not surprising, since in vitro differentiated cardiomyocytes from stem cells have been widely shown to have a morphologically and electrophysiologically immature cardiac phenotype [12, 24, 25]. It is likely that W8B2^+^ CSC-derived cardiomyocytes also have an immature phenotype given the nuclear localization of contractile proteins, which is in line with several studies showing the expression of contractile structures (such as tropomyosin and troponin) in the nucleus of rat mesenchymal stem cells (MSCs) at an early stage of differentiation [26].

Other cardiac markers such as GATA5 and GATA6 transcription factors were also overexpressed during W8B2^+^ CSC cardiac differentiation. These two factors play a fundamental role in cardiac morphogenesis [27, 28], particularly in relation to the development of a nodal phenotype. Similarly, Iachininoto et al. [29] reported that GATA6 is also overexpressed during the cardiac differentiation of c-kit^+^ human umbilical cord cells, as well as during the reprogramming of human MSCs to cardiovascular precursors [30]. No induction of GATA4 was detected at the transcriptomic scale in our model, which is in agreement with two other studies [12, 13]. The present results also highlight upregulated expression of BNP during W8B2^+^ CSC differentiation, an effect that was also found during cardiomyocyte differentiation of rat MSCs and monkey embryonic stem cells [31–33]. BNP is able to induce cardiomyocyte differentiation of mouse CSCs [34] and improve cardiac function following transplantation of MSCs into a mouse model of heart failure [35]. Recently, Al-Maqtari and colleagues reported that BNP-encoding transcripts were overexpressed when human c-kit^+^ CSCs were induced by some cardiac transcription factors [36]. In contrast to this, we did not observe any changes in the expression of transcripts coding for atrial natriuretic peptide (ANP; Fig. 1), which plays an important role in the differentiation of rat fibroblasts into myofibroblasts [37, 38]. The expression and role of these two types of natriuretic peptides may be cell-type dependent. Taken together, these results confirm the immature phenotype of cardiomyocytes obtained after the in vitro differentiation of W8B2^+^ CSCs.

### Transcriptomic profile of ion channels and calcium oscillations during in vitro cardiac differentiation of W8B2^+^ CSCs

Our results showed transcriptomic induction of the Cav 1.3 and Cav1.2 calcium channels (underlying the ICa,L current). Cav1.3-mediated ICa,L began to be activated about midway between the maximum diastolic potential (of cardiac pacemaker activity) and the threshold of the following AP upstroke, while the Cav1.2-mediated ICa,L was activated during the AP upstroke [39–41]. Other transcripts encoding for the Cav3.2 calcium channel, which is responsible for the ICaT current (implicated in the slow depolarization phase of the pacemaker activity of the sinoatrial node), were induced after W8B2^+^ CSC differentiation. The overexpression of these calcium channels (L and T type) is correlated with that of the regulatory subunits important for the modulation of their activity. It was observed previously that transcripts encoding Cav1.2 calcium channels were induced, while transcripts encoding Cav3.2 channels showed no variation during myogenic differentiation of murine MSCs [42]. These calcium currents could play an important role in the regulation of the cellular properties of stem cells. Indeed, Hotchkiss and colleagues demonstrated that the inhibition of L-type calcium channels by nifedipine decreased the proliferation and cardiac differentiation of mouse CSCs [16].

The in vitro cardiac differentiation of our W8B2^+^ CSCs was also accompanied by the transcriptomic induction of HCN1 (neuronal isoform), HCN4 (cardiac isoform), HCN2 and HCN3. These HCN channels, particularly HCN4, are responsible for the currents underlying the cardiac rhythmic activity of nodal cells. Several studies on human MSCs and other species have clearly shown that functional pacemaker differentiation correlated with gene overexpression of the HCN1 or HCN4 isoforms [43–45]. Overall, the analysis of transcripts coding for ion channels after the differentiation of W8B2^+^ CSCs may indicate an orientation towards a pacemaker cell phenotype (by the induction of HCN, and ICa,T and ICa,L isoform CaV1.3).

In addition to the overexpression of transcripts coding for calcium channels (L and T type) after in vitro differentiation of W8B2^+^ CSCs, our results also indicated a change in the expression of players regulating calcium homeostasis and which are involved in excitation–contraction coupling. Indeed, we observed transcriptional induction of SERCA, NCX exchanger and IP3 receptors, and strong downregulation of CALM3.

On the other hand, the calcium homeostasis-related gene expression levels are similar between the W8B2 human cardiac stem cells and the human induced pluripotent stem cells differentiated into cardiomyocytes (hiPSC-CMs) (see Additional file 1, Fig. S1). Note the very low expression of RYR2 and CasQ2 genes in human W8B2 CSC^+^ compared to hiPSC-CMs, suggesting an important role of IP receptors in the regulation of calcium homeostasis of differentiated W8B2^+^ cells.

In addition, calcium imaging revealed significant changes in the frequency, peak amplitude, duration and TTP of calcium oscillations observed during the cardiac differentiation process. Pharmacological experiments carried out on differentiated W8B2^+^ CSCs revealed a major role of the NCX exchanger in the generation of these oscillations. The results of pharmacological inhibition suggested that intracellular calcium release is generated by L-type Cav calcium channels and IP3 receptors, while calcium reuptake is controlled by SERCA. In previous reports, calcium oscillations in stem cells were recorded and analyzed mainly in human MSCs [46, 47], but they have also been observed and characterized in other cell types such as human cardiac fibroblasts [48], human aortic and cerebrovascular endothelial cells [49, 50] and lymphocytes [51]. However, their roles in these different cell types remain to be elucidated. It is also important to point out the lack of data on the evolution and role of these calcium oscillations during the cardiac differentiation process. During the in vitro cardiac differentiation of W8B2^+^ CSCs, we observed a decrease in the frequency and an increase in the peak amplitude of calcium oscillations. These slow oscillations became very regular, indicating the emergence of a calcium clock which seems to play an important role in the process of differentiation. A study on the osteogenic differentiation of human MSCs also reported a decrease in the frequency of calcium oscillations during differentiation but no change in peak amplitude [52]. Interestingly, the authors of that study stated that the osteogenic differentiation of MSCs combined with the reduction of calcium oscillation frequency increased the differentiation efficiency. Calcium oscillations are also present in rat liver stem cells and human MSCs when they are cultured with neonatal rat cardiomyocytes [53, 54]. These oscillations were synchronous with those of the cardiomyocytes and provoked the acquisition of a cardiac phenotype along with the transcriptomic induction of cardiac genes. It has been suggested that the frequency of calcium oscillations in excitable and non-excitable cells regulates gene expression differently and that only NF-kB responded to low frequency oscillations [55]. The present results highlighted significant under-expression of transcripts coding for CaM3, during the cardiac differentiation process. Furthermore, no change in the transcriptional expression of calcineurin was detected. Both CaM and calcineurin are the main mediators of the molecular effects of calcium, achieving their action by activating several molecular targets such as NF-AT, NF-KB, and CaMKII. The calcineurin-activated NFAT transcription factor may act as a decoder for calcium oscillations [55]. For example, in human umbilical cord vein endothelial cells, the frequency, peak amplitude and duration of calcium oscillations regulate the activity of the NF-κB transcription factor [56], which is only activated when the peak amplitude of the oscillations reaches a threshold value. Thereafter, NF-κB activity is more intense as the frequency and/or the duration of the oscillations increase.

Several studies have also highlighted that the frequency and duration of oscillations regulate the activity of NFAT in several cell models [57–59]. A recent study used optogenetic calcium oscillation control to show that the NFAT transcription factor is also sensitive to variations in the measured area under the curve (AUC) of calcium oscillations [60]. The available data show that NFAT is activated by calcium oscillations with a frequency of 1–1000 MHz and a duration of 0.2–50 s [61]. The activation of NF-KB by calcineurin is also known to be sensitive to the frequency and duration of calcium oscillations in several cell types [50, 62–65]. NF-KB is activated by calcium oscillations with a frequency of 3 to 10 MHz and a duration of 36–52 s [61]. Likewise, the activation of type II calcium calmodulin kinase (CaMKII) is sensitive to calcium oscillations with a frequency of 100–4000 MHz and a duration of 200 ms [61, 66, 67]. Calpain protease was also considered to be a calcium decoder [68] and is activated by calcium oscillations with a frequency of 1–50 Hz and a duration of 20 ms [61]. During the differentiation of stem cells into cardiomyocytes, the mitogen-activated protein kinase pathway (MAPK) plays a central role [69–71] and is also sensitive to calcium oscillations [72–74]. Activation of this pathway (via ERK phosphorylation) is regulated by calcium oscillations at a frequency of 1.7–17 MHz and an approximate duration of 50 s [75]. These results were confirmed in a theoretical modeling study showing a negative correlation between the decrease of oscillatory frequency and activation of the MAPK pathway [76]. It is highly likely that changes in calcium oscillation parameters (frequency, peak amplitude, duration, …) observed after the differentiation of W8B2^+^ CSCs in the present study are related to the acquisition of the observed immature cardiac phenotype.

Further to the above, changes in the calcium oscillation parameters may also act on molecular players to modulate the expression of cardiac genes. According to the literature, we speculate that the MAPK pathway could be a potential decoder of changes in calcium oscillation parameters observed in differentiated W8B2^+^ CSCs. Under our experimental conditions, the frequency (5 MHz) and duration (92 s) of calcium oscillations were close to those where MAPK is sensitive [61]. In addition, we observed a decrease in the calcium oscillatory frequency during cardiac differentiation, which was similar to the theoretical modeling study in which the decrease in oscillatory frequency was related to activation of the MAPK pathway [76]. According to Smedler, MAPK in cardiomyocytes is also sensitive to oscillations, especially since this pathway is involved in cardiac differentiation [61]. It is likely, however, that the NFAT and NF-KB (calcineurin-regulated) transcription factors and CaMKII are not the decoders of calcium oscillations observed in differentiated W8B2^+^ CSCs because the transcriptional expression of calcineurin and calmodulin did not change and/or was under-expressed. In addition, calpain and CaMKII are not sensitive to the frequency and duration observed in differentiated W8B2^+^ CSCs.

It is important to note that, in addition to its essential role in the proliferation and self-renewal of W8B2^+^ CSCs [15], the calcium-activated potassium channel BKCa appears to play a role in differentiation. Inhibition of the BKCa channel drastically decreased the calcium oscillation parameters (frequency, peak amplitude, duration, etc.) observed in differentiated W8B2^+^ CSCs. On the other hand, spontaneous calcium oscillations induce fluctuations in the membrane potential via the IKCa potassium channel [47] and are associated with the nuclear translocation of NFAT [46]. Another study has shown that the membrane potential regulates IP3-controlled calcium oscillations [77], suggesting involvement of the BKCa channel in the regulation of these oscillations.

## Conclusions

Transcriptomic analysis showed that W8B2^+^ CSCs can progress towards an immature nodal phenotype when they are differentiated in vitro. This differentiation is accompanied by significant changes in calcium activity and can be linked to the appearance of regular calcium oscillations. Our experiments showed that in vitro differentiation, as for induced pluripotent stem cell differentiation, can lead to the production of typical immature cardiomyocytes. We also found that in vitro differentiation using the method of Zhang and colleagues does not seem to be the best way to induce a mature cardiac phenotype. Overall, this work provides important insights into the physiology of cardiac stem cells, including the involvement of ion channels and dynamic calcium activity during differentiation. Further work should concentrate on a protein scale analysis of ion channels and players in calcium homeostasis, additional study of the spontaneous calcium activity in undifferentiated W8B2^+^ CSCs by pharmacological characterization, and identification at the molecular level of the targets involved in the modification of calcium oscillation parameters during W8B2^+^ CSC differentiation.

## Supplementary information

**Additional file 1: Figure S1.** Radar plot of calcium homeostasis-related and HCN gene expression level. Expression level of each gene versus reference gene (RPL13A) in differentiated W8B2 CSC+ cells is depicted in blue (n = 6). As a reference, in orange, the expression level of the same genes in human induced pluripotent stem cells differentiated into cardiomyocytes (hiPSC-CMs) generated from four different healthy donors (number of differentiations = 12) is depicted (ref. Al Sayed ZR et al., Cardiovascular Research, 2020). Data are in logarithmic scale (log2).

## Data Availability

The data in this study are available from the author for correspondence upon reasonable request.

## Abbreviations

a-actinin: Actinin alpha
ANKB: Ankyrin B
ANP: Atrial natriuretic factor
BKCa: Large conductance calcium-activated potassium channel
BNP: Brain natriuretic peptide
b-MHC: Myosin heavy chain beta
CaMKII: Type II calcium calmodulin kinase
CALM1: Calmodulin 1
CALM3: Calmodulin 3
CAM-PRP: Calmodulin-dependent protein phosphatase
CASQ2: Calsequestrin 2
Cav: Calcium channel, voltage-dependent
Cav1.2: Calcium channel, voltage-dependent, L type, alpha 1C subunit
Cav1.3: Calcium channel, voltage-dependent, L type, alpha 1D subunit
Cav3.1: Calcium voltage-gated channel subunit alpha1 G
Cav3.2: Calcium channel, voltage-dependent, T type, alpha 1H subunit
Cava2d2: Voltage-dependent Ca^2^^+^ channel Cav alpha2delta2 subunit
cDNA: Complementary DNA
c-kit+ CSCs: Cardiac stem cells expressing c-kit marker
CLASP2: Cytoplasmic linker associated protein 2
CSCs: Cardiac stem cells
Ct: Threshold cycle
EGM-2: Endothelial cell growth medium 2
ERK: Extracellular signal-related kinase
FACS: Fluorescence activated cell sorting
GATA-3: GATA-binding factor 3
GATA-4: GATA-binding factor 4
GATA-5: GATA-binding factor 5
GATA-6: GATA-binding factor 6
GCaMP: Genetically encoded calcium indicator consisting of circularly permuted GFP (cpGFP), the calcium-binding protein calmodulin (CaM), and CaM-interacting M13 peptide
GPD1L: Glycerol-3-phosphate dehydrogenase 1 like
HCN: Hyperpolarization-activated cyclic nucleotide gated
HCN1: Hyperpolarization activated cyclic nucleotide gated potassium and sodium channel 1
HCN2: Hyperpolarization activated cyclic nucleotide gated potassium and sodium channel 2
HCN3: Hyperpolarization activated cyclic nucleotide gated potassium and sodium channel 3
HCN4: Hyperpolarization activated cyclic nucleotide gated potassium and sodium channel 4
hEag1: Human Ether à Go-Go 1
HEY2: Hes related family BHLH transcription factor with YRPW Motif 2
ICa.L: L-type calcium current
ICa.T: T-type calcium current
IMDM medium: Iscove's modified Dulbecco's medium
IP3: Inositol trisphosphate
IRX4: Iroquois homeobox 4
IRX5: Iroquois homeobox 5
IRX3: Iroquois homeobox 3
Isl-1+CSCs: Cardiac stem cells expressing Isl-1 marker
ITPR3: Inositol trisphosphate receptor 3
KV: Voltage-gated potassium channels
MAPK: Mitogen-activated protein kinase pathway
MEF2C: Myocyte enhancer factor 2C
MSCA-1: Mesenchymal stem cell antigen-1
MYL2: Myosin light chain 2
MYL7: Myosin light chain 7
MYH6: Myosin heavy chain 6
MYH7: Myosin heavy chain 7
M199: Medium 199
NCX: Sodium-calcium exchanger
NCX1: Sodium-calcium exchanger 1
NF-AT: Nuclear factor of activated T-cells
NF-KB: Nuclear factor-kappa B
Nkx2.5: NK2 homeobox 5
PE: Phycoerythrin
PLN: Phospholamban
RPL13A: Ribosomal protein L13a
RRAD: Ras related glycolysis inhibitor and calcium channel regulator
RT-PCR: Reverse transcription‐polymerase chain reaction
RyR: Ryanodine receptor
RYR2: Ryanodine receptor 2
Sca-1 + CSCs: Cardiac stem cells expressing Sca-1 marker
SERCA2: Sarco-endoplasmic reticulum calcium pumps 2
SERCA3: Sarco-endoplasmic reticulum calcium pumps 3
SP: Side population
TBX2: T-box transcription factor 2
TBX3: T-box transcription factor 3
TLDA: Taqman low-density array
TNAP: Tissue non-specific alkaline phosphatase
TNNI3: Troponin I type 3
TNNT2: Troponin T2
W8B2 ^+^ CSCs: Cardiac stem cells expressing W8B2 marker
[Ca^2 +^]i: Changes in intracellular calcium
