# Supplementary figures and images for: In vitro differentiation of W8B2+ human cardiac stem cells: gene expression of ionic channels and spontaneous calcium activity
Source: Cell Mol Biol Lett. 2020 Nov 5;25:50. doi: 10.1186/s11658-020-00242-9 (PMC7646077; doi:10.1186/s11658-020-00242-9)

Expression level versus reference gene (log2)

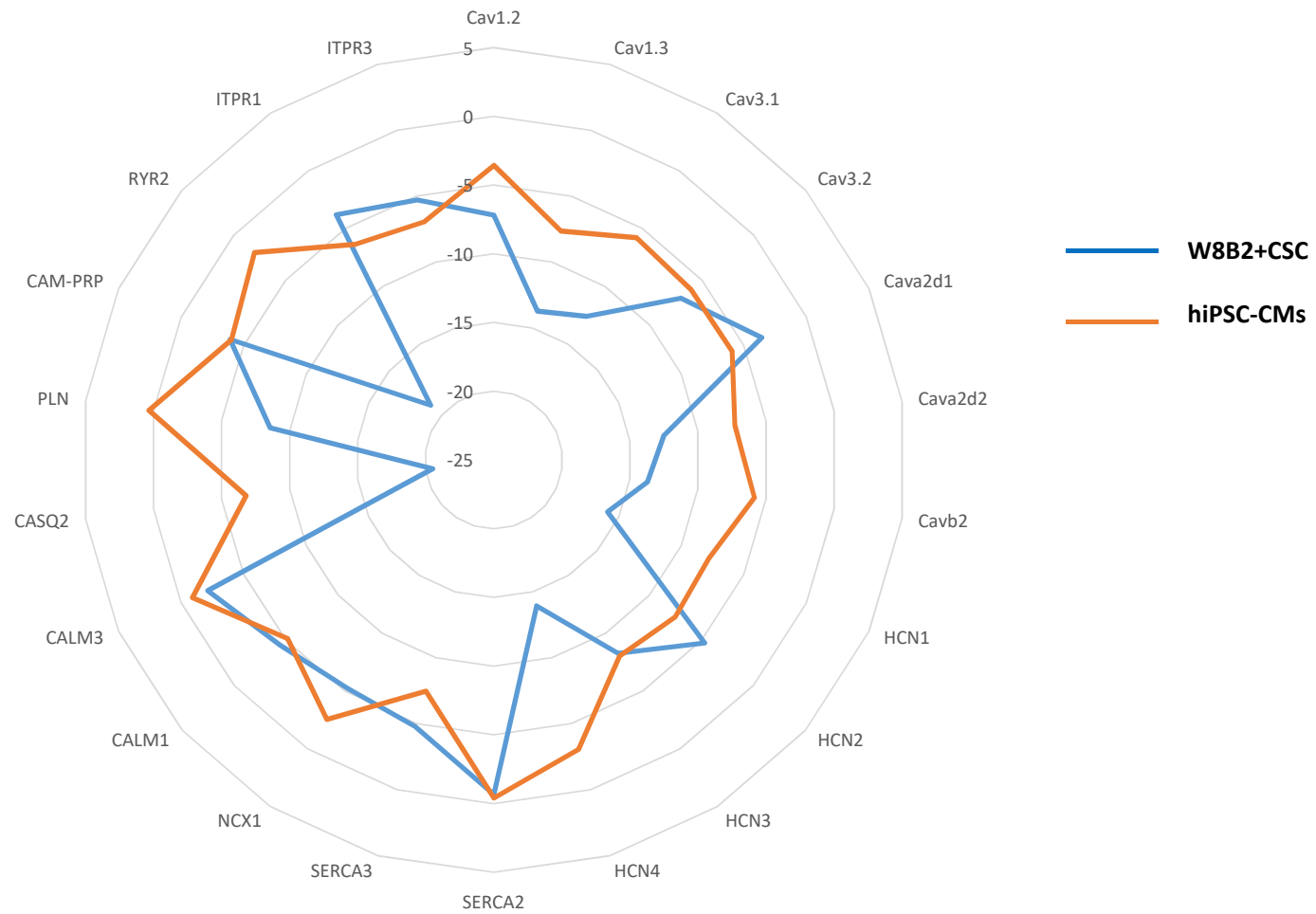

Supplement: Supplementary file 1 — Additional file 1: Figure S1. Radar plot of calcium homeostasis-related and HCN gene expression level. Expression level of each gene versus reference gene (RPL13A) in differentiated W8B2 CSC+ cells is depicted in blue (n = 6). As a reference, in orange, the expression level of the same genes in human induced pluripotent stem cells differentiated into cardiomyocytes (hiPSC-CMs) generated from four different healthy donors (number of differentiations = 12) is depicted (ref. Al Sayed ZR et al., Cardiovascular Research, 2020). Data are in logarithmic scale (log2). [file 11658_2020_242_MOESM1_ESM.pdf]
